# Perspective: Challenges for Personalized Nutrition in the Current United States Regulatory Framework and Future Opportunities

**DOI:** 10.1016/j.advnut.2025.100382

**Published:** 2025-01-25

**Authors:** Sharon M Donovan, Mariette Abrahams, Joshua C Anthony, Robert Bergia, Gil Blander, Tristin D Brisbois, Anna-Sigrid Keck, Edwin G Moore, Timothy A Morck, Kristin M Nieman, Jose M Ordovas, Alison Steiber, Barbara L Winters, Thuyvan Wu

**Affiliations:** 1 Department of Food Science and Human Nutrition, and Personalized Nutrition Initiative, Carl R. Woese Institute for Genomic Biology, University of Illinois Urbana-Champaign, Urbana, IL, United States; 2 Qina, Ltd, Olhao, Portugal; 3 Nlumn, LLC, Princeton, NJ, United States; 4 Archer Daniels Midland, Decatur, IL, United States; 5 Segterra (Inside Tracker), Cambridge, MA, United States; 6 Advanced Personalization Ideation Center, PepsiCo Inc, Purchase, New York, NY, United States; 7 Personalized Nutrition Initiative, University of Illinois Urbana-Champaign, Urbana, IL, United States; 8 Technology Entrepreneur Center, University of Illinois Urbana-Champaign, Urbana, IL; 9 Spectrum Nutrition, LLC, Saint Cloud, FL, United States; 10 Friedman School of Nutrition Science and Policy and Nutrition and Genomics Laboratory, Jean Mayer-United States Department of Agriculture Human Nutrition Research Center on Aging, Tufts University, Boston, MA, United States; 11 Academy of Nutrition and Dietetics, Chicago, IL and Washington, DC, United States; 12 Pharmavite, LLC, West Hills, CA, United States; 13 BioPhia Consulting Inc, Lake Forest, IL, United States

**Keywords:** precision nutrition, regulation, data privacy, nutrigenomics, evidence-based practice, nutrition recommendations, dietary guidelines, biomarkers, wearable technology, artificial intelligence

## Abstract

Personalized nutrition (PN) aims to provide tailored dietary recommendations to improve a person’s health outcomes by integrating a multitude of individual-level information and supporting desired behavior changes. The field is rapidly evolving with technological advances. As new biomarkers are discovered, wearables and other devices can now provide up-to-the-minute insights, and artificial intelligence and machine learning models support recommendations and lifestyle behavior change. Advancements in the field enhance the potential for tailored and effective programs but raise important questions regarding user safety, security, health, transparency, and privacy. The Personalized Nutrition Initiative at the University of Illinois held the workshop, “Challenges for Personalized Nutrition in the Current United States Regulatory Framework and Future Opportunities,” to address regulatory implications of current PN programs, future innovation within the current framework, and existing complexities of oversight. A majority of PN programs combine multiple components, and these components may need to be regulated differently. The areas of food, supplements, in vitro diagnostics, and medical and wellness devices were described and discussed as they apply to PN programs. The speakers and discussants concluded that regulatory guidance for PN programs should focus on ensuring *1*) safety and accuracy of the tests and devices, *2*) credentialed and skilled experts develop the advice, *3*) responsible and clear communication of information and benefits, *4*) substantiation of scientific claims, and *5*) procedures are implemented to protect user privacy. Furthermore, as this field incorporates new devices, biomarkers, behavior-based tools, and the integration of artificial intelligence and machine learning, the need to adapt the existing regulatory framework was also considered. Working closely with regulatory bodies is required and should be an opportunity to provide users with transparency, build trust, and create a source of differentiation for PN innovators.

**Statements of significance**

Innovations in the personalized nutrition (PN) field promise to improve opportunities to deliver tailored and effective programs but raise important questions regarding regulatory oversight since current products and services include components that are regulated by different offices in the Food and Drug Administration. The Personalized Nutrition Initiative at the University of Illinois, Urbana, organized a workshop to address the regulatory implications of current PN products, future innovation within the current framework, and existing complexities of oversight.

## Introduction

Nutrition recommendations and dietary guidelines can be effective when they are evidence-based, applicable to, and adopted by a specific population, subpopulation, or individual. The USDA and Health and Human Services Dietary Guidelines for Americans (DGAs) provide population-based nutrition recommendations to meet nutrient needs, promote health, and prevent chronic disease [1]. The definitions of precision nutrition and personalized nutrition (PN) are not standardized and often overlap in this rapidly evolving field [2]; however, for the purposes of this report, the following definitions were utilized. Precision nutrition provides tailored dietary recommendations for specific subgroups within the general population. These groupings can be based on demographic factors (e.g., age, sex), biological markers (e.g., genetics, blood biomarkers), behavioral patterns, environmental factors, or risk for certain diseases (e.g., type 2 diabetes). The goal is to provide more targeted advice than general population guidelines but not as individualized as PN. Information used for recommendations should be centered on evidence-based science and designed to promote dietary behavior change by considering unique goals and preferences. PN focuses on the individual level, considering unique genetic, phenotypic, medical, and lifestyle information to tailor dietary recommendations. The aim is to promote dietary behavior changes by aligning them with individual goals and preferences.

PN and precision nutrition often incorporate an interdisciplinary approach, relying on nutrition, systems biology, and behavioral sciences to develop tailored recommendations targeting a quantifiable improvement in health or function [3,4]. Approaches should be structured within sound dietary guidance to help support individual needs while achieving public health recommendations. Indeed, emerging research suggests that PN, which combines systems biology with behavioral sciences, may help address an individual’s preferences and unique health needs and achieve public health recommendations more effectively than general guidelines [[5], [6], [7], [8], [9], [10]]. As other findings, primarily focused on genetic-driven personalized interventions, have been less effective, this area requires further research [11,12].

The increasing consumer demand for tailored health interventions underscores the importance of developing scientifically robust, inclusive, and scalable PN programs [13,14]. Furthermore, the field of PN is rapidly incorporating new tools to measure biomarkers and integrating artificial intelligence (AI) and machine learning (ML) models to support recommendations and lifestyle behavior change. These tools hold promise to improve our understanding of health requirements and scale the PN field by making it more accessible, but they also raise important questions regarding user safety, security, health, and privacy.

As the PN field grows, it is critical to consider the regulatory framework to establish and maintain credibility, build user trust and engagement, and prevent misinformation. Guidance areas for PN programs include ensuring the safety and accuracy of the tests and devices, having credentialed and skilled experts that develop and deliver the advice, responsible and clear communication of information and benefits, and scientific substantiation of claims [15]. Therefore, on 6 March, 2024, the Personalized Nutrition Initiative at the University of Illinois Urbana-Champaign held a virtual workshop, “Challenges for Personalized Nutrition in the Current United States Regulatory Framework and Future Opportunities.” The workshop goals centered around the current regulatory framework for PN and included understanding *1*) the PN process and regulatory implications, *2*) the history, present status, applications, and limitations of the current United States regulatory framework when applied to PN, and *3*) the complexities of oversight of PN programs and the future horizon. The main purpose was to better understand current regulatory guidelines and how PN innovation fits within them to help guide responsible program development. Working with this framework will also benefit users by supporting safety, consistency, and credibility for PN programs. At the same time, rapidly developing technologies, such as AI consumer wearables and continuous biosensors, are changing approaches to PN and health. Accordingly, this workshop also considered whether new regulations are needed to oversee this changing landscape.

### Understanding the PN system and regulatory implications

#### The PN system

**FIGURE 1:**
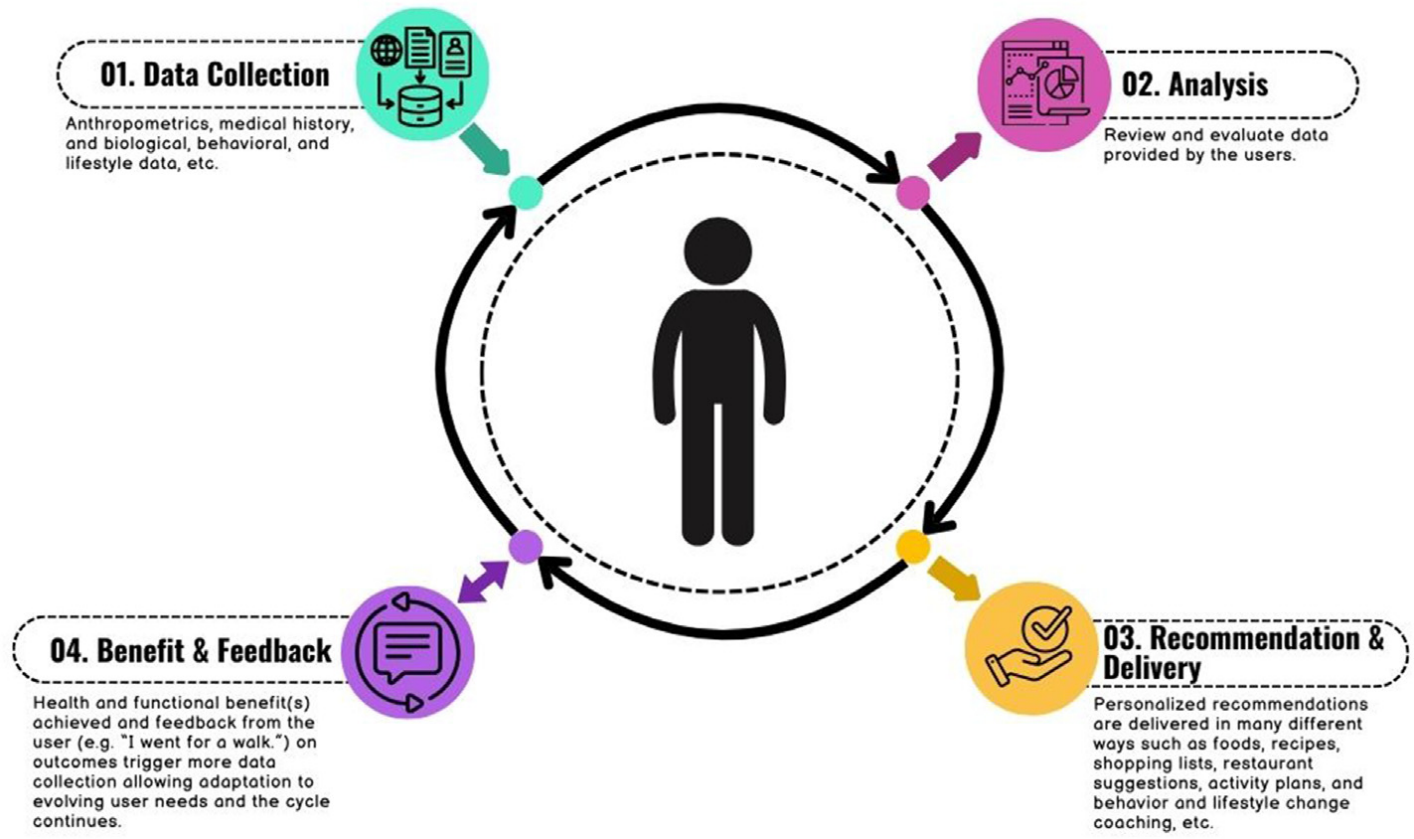
The personalized nutrition system. Created using canva.com.

The PN system (Figure 1) includes health and biological data, social and behavioral data, and consumer purchasing data collection and analysis, delivery of recommendations to the user, assessment progress, and ongoing recommendation adjustment, which continues the cycle, aimed at improving health outcomes [16]. Adapting recommendations to meet individual needs is key, as health and functional goals can change over time. Each step in this system has implications regarding regulations and privacy and varies depending on the target group and the composition of the solution.

#### Data collection and analysis

**FIGURE 2:**
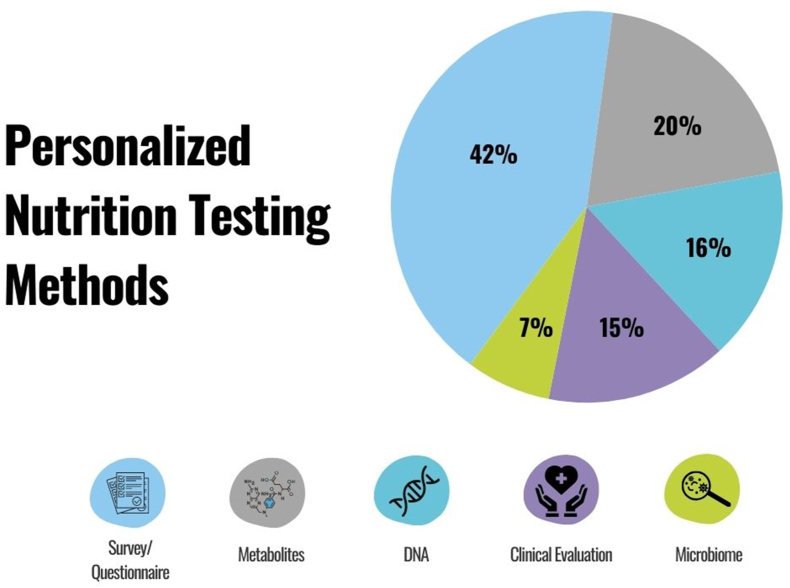
Common testing/data collection methods that companies offer in personalized nutrition. Created using canva.com. DNA, deoxyribonucleic acid. Reproduced from source: Qina database [17] with permission.

The first stage in the PN system is data collection. This can include anthropometric measurements, medical history, other biological data (e.g., genetics, blood biomarkers, and microbiome), behavioral data, lifestyle data, and environmental data. Regulatory considerations depend on the type of data that is collected, how it is collected, managed, and stored, and how the information is used. Surveys completed by the individual (e.g., food preferences/avoidances, nutrient intake, barriers to access, and quality of life) are often used to support personalized recommendations (Figure 2) [17]. If the data are considered medical information [18], it may be subject to the Health Insurance Portability and Accountability Act of 1996 [19]. If individuals provide information on a medical diagnosis, medications, or even family medical history, this becomes an area of regulatory ambiguity. The recommendations provided may be considered medical nutrition therapy (MNT) [20]. MNT is a nutrition-based therapy for many short- and long-term health conditions and requires a physician’s referral. MNT includes conditions such as gastrointestinal, cardiovascular, and metabolic disorders and is subject to further regulation. When data collection is limited to adults (18 y and older), sex, and markers of wellness (e.g., nutrient status and activity level) that lifestyle changes can modify, the program may not require medical oversight. If the recommendations using this data are not designed to diagnose, cure, mitigate, treat, or prevent disease, then medical oversight may not be required [[18], [19], [20]].

#### Recommendations and benefits to improve health outcomes

**FIGURE 3:**
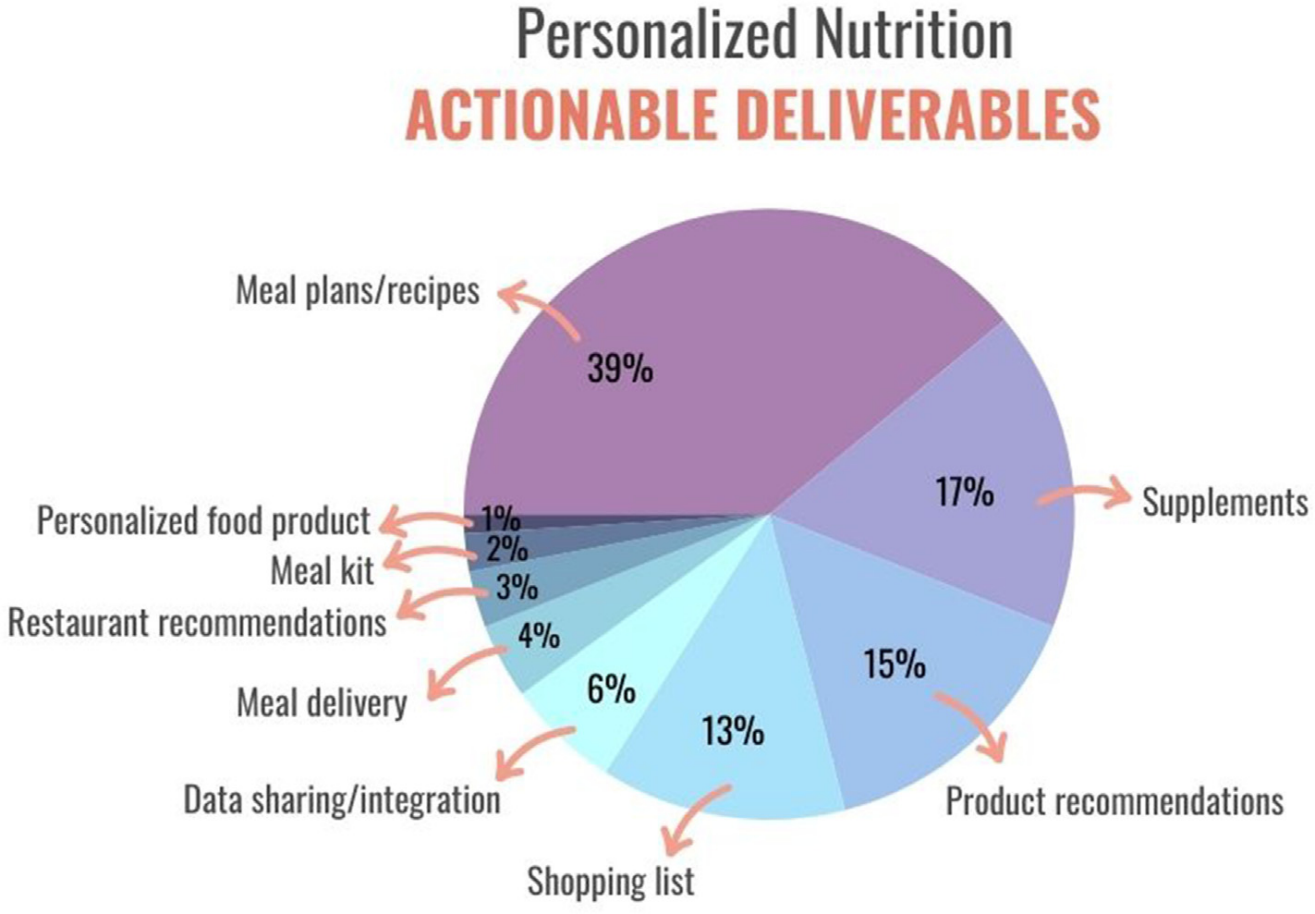
Common actionable deliverables provided by personalized nutrition programs. Created using canva.com. Reproduced from source: Qina database [17] with permission.

Once data are collected and analyzed, individuals are provided with personalized recommendations, including actions for quantitative improvements in their health. The recommendations can differ, especially if the data are integrated with other platforms. Deliverables may include supplements, personalized foods, meal plans or kits, recipes, shopping lists, restaurants, activities, and other lifestyle practices (Figure 3) [17]. Companies must follow guidance and regulations based on the personalized approaches and interventions offered. For example, food has specific labeling [1,21] and claim guidance and regulations [22]. Supplements have detailed regulations [23] on ingredient labeling and claims. Medical foods [24], foods for special dietary use (FSDU) [25], and functional food claims [26] have specific guidance and regulations set by the Food and Drug Administration (FDA), the Federal Trade Commission (FTC), and the USDA. Although the intent of this article is to focus on PN in the United States, there are resources available for other regions of the world on this topic [[26], [27], [28]].

Behavior change techniques, rooted in established psychological theories, are integral to improving health outcomes in PN programs and require design by appropriate experts and oversight by credentialed and skilled experts [3,29]. Although health professionals are not always integrated into PN programs, there are important regulatory and clinical reasons for doing so. First, they can help provide the necessary expertise to develop the program and monitor and analyze data to assess behavior change and health outcomes. Next, PN participants need support to understand their data, recommendations, and actions to support lifestyle behavior change and avoid confusion that can lead to program discontinuation. For this reason, coaching and/or counseling are included in some programs to help augment motivation and success in improving health and wellness. It is important to understand that there is a wide variety of expert types and skill sets that may provide behavior change and nutritional guidance in the wellness and lifestyle space. They may hold an array of titles and have training experiences that range in length from weeks to years. Globally, registered dietitians are formally trained and regulated food and nutrition practitioners. In the United States, registered dietitian nutritionists (RDNs) have completed a minimum of a master’s degree (effective 1 January 2024) through an Accredited Academy of Nutrition and Dietetics program [30] and a supervised practice requirement, passed the National Board examination, and continue professional development throughout their careers [31]. There are state and national regulations for RDNs. In addition to RDNs providing nutrition and lifestyle guidance in PN programs, there are important leadership and collaboration opportunities for credentialed experts to provide support to change lifestyle behaviors (e.g., physicians, nurses, psychologists, behavioralists, health and wellness coaches, and other clinicians etc.).

There is also a distinction between those who can order, interpret, provide ongoing monitoring, and provide more complex nutrition science-based recommendations for medical conditions and those who should only operate within the wellness and lifestyle space. Some PN programs address collecting medically linked health data by having licensed healthcare professionals provide scientific oversight and clinically based standards of practice in the state where users reside and oversee the ordering, interpretation, and follow-up of any tests. When this process is in place, it helps to alleviate the risk of misinformation, no information, and/or inaccurate guidance. Companies that provide these services will often seek to provide transparency and mitigate risk by highlighting that the information provided is not intended to treat, diagnose, mitigate, prevent, or cure diseases. Claims made must be substantiated by appropriate scientific evidence and meet FDA and FTC standards. In addition, benefits should be communicated in the context of health as opposed to disease (e.g., “helps support heart health” rather than “prevents cardiovascular disease”). Benefit statements are still subject to regulations, and companies may be held accountable if they use medically linked health data to manage medically related conditions. Credentialed researchers and healthcare professionals should oversee and approve the scientific substantiation and accuracy of the algorithms and claims. When the data collected and subsequent health and behavior change recommendations provided fall under MNT, or if the data captured are indicative of disease risk, then a physician referral is required, and nutrition guidance is provided by an RDN.

### History, present status, applications, and limitations of the current United States regulatory framework when applied to PN

The FDA and FTC are the major regulatory bodies overseeing PN programs. The mission of the FDA [32] is to protect public health by ensuring the safety, efficacy, and security of the human food supply, biological products, medical devices, drugs, cosmetics, and products that emit radiation. The mission of the FTC is to protect the public from deceptive or unfair business practices and unfair competition methods through law enforcement, advocacy, research, and education. The FDA and the FTC [33] work in tandem to protect public health by sharing jurisdiction over health-related products (e.g., foods, beverages, supplements). The FDA is responsible for regulating product labeling and claims, whereas the FTC is responsible for enforcing laws against misleading advertising by ensuring truthful and substantiated health-related product labeling and marketing. The following sections consider how nutrition and healthcare developments have shaped current regulations, how the PN system fits within the current regulatory framework, and some of the challenges and opportunities relative to future innovations in PN.

#### Human food program

The FDA’s legal authority for regulation of general food, FSDU, dietary supplements, infant formulas, and medical foods is the Food, Drug, and Cosmetic Act and is overseen by the Human Food Program (HFP; formerly the Center for Food Safety and Applied Nutrition) [[34], [35], [36]] (Table 1) [23,24,25,37]. Perhaps 1 of the earliest examples of a product designed based on genetic variation came in the 1950s with the development of the infant formula, Lofenalac (Mead Johnson). It was specifically formulated for infants with inborn errors in metabolism and phenylketonuria. These infants require medical food for normal growth and development.

**TABLE 1:** Food categories regulated by the Center for Food Safety and Applied Nutrition.

Food category	Description
Dietary supplements	Products (vitamins, minerals, herbs, other botanicals, amino acids, dietary substances to increase intake, or a combination) intended to supplement the diet that is not represented as a conventional food or sole meal/diet item.^1^
Foods for special dietary use (FSDU)	Foods are distinguished from general foods based on supplying particular dietary needs due to health conditions or age or supplementing or fortifying the usual diet with other dietary properties.^2^
Infant formula	Infant formula has its own set of regulations outside of foods and supplements.^3^
Medical foods	Used to manage the diet of a patient who has impaired ability to metabolize certain foods and must meet all 5 FDA criteria for a medical food.^4^

Abbreviation: FDA, Food and Drug Administration.

1 As defined by the Dietary Supplement Health and Education Act [23].

2 FDA criteria for FSDU [25].

3 FDA regulations on infant formula [37].

4 FDA criteria for a medical food [24].

In 1990, the Nutrition Labeling and Education Act exempted medical foods from certain nutrition labeling requirements, health claims, and nutrient content claim requirements that most other foods had to abide by. Medical foods are exempt from the requirements for health claims that apply to foods for the general population but nonetheless are allowed to name a disease or condition in labeling without being considered a drug [38]. The FDA has clear boundaries between food and drugs. A food is not permitted to claim to diagnose, treat, prevent, mitigate, or cure a disease or symptoms of a disease. Therefore, the wording is precise: medical foods are not for managing the disease but “…intended for the dietary management of a patient, who because of therapeutic or chronic medical needs…” has a unique nutritional need that differs from the general population. The FDA identified 5 criteria that must be met to qualify a food for this labeling exemption [24,39]. The objective of a specially formulated medical food is to help manage the diet of a patient with a disease or condition that imposes distinctive nutritional requirements, which cannot be achieved by the modification of the normal diet alone. A healthcare professional or team needs to be actively involved in the MNT process and evaluate the use and dose of the medical food. Other components under the HFP include general foods, prepared foods, and FSDU. FSDU is a regulated category of food. The definition of FSDU can be found in 21CFR105.3(a) [25] (Table 1).

The term functional foods can be confused with medical foods or FSDU. Functional foods are not an FDA-defined or regulated category of food. The term is used in common language and applies to several foods that may be specially formulated or enriched with phytonutrients or other bioactives that may have metabolic or physiologic value. Functional food claims, as with all foods that have any health claim (qualified or authorized), structure-function claim, or nutrient content claim attached, require substantiation, be truthful and not misleading, and must meet FDA and FTC criteria [22,40]. All foods must meet safety, labeling, manufacturing, and marketing regulations. FTC has jurisdiction over advertising content and claims and requires documentation or substantiation of claims based on reputable scientific studies. There are other food categories regulated by the FDA’s HFP. For example, infant formulas are a unique category that requires premarket notification. Infant formulas have their own set of regulations that can be reviewed elsewhere [37]. A thorough review of food claims is outside the scope of this article, and the details can also be reviewed elsewhere [41,42].

#### Dietary supplements and PN

The HFP is also responsible for regulating dietary supplements under the Dietary Supplement Health and Education Act (DSHEA) [23]. The 2 primary goals of DSHEA are to provide consumers continuous access to a wide variety of dietary supplements and information on their intended use. The provisions of DSHEA include defining dietary supplements and ingredients, the process for introducing new dietary ingredients (NDI), and the requirements for statements of nutrition support. The DSHEA also authorized good manufacturing practices for supplements and established labeling requirements and exemptions, which expanded the safety standard.

Dietary supplements are defined by the FDA following DSHEA [23] (Table 1). The FDA confirms that experts have evaluated the safety of a dietary supplement ingredient through the generally recognized as safe or the NDI notification regulations under DSHEA. Generally recognized as safe substances, they are considered safe by qualified experts under the conditions of their intended use [43]. Dietary ingredients used before 15 October 15, 1994, are “grandfathered in” as accepted safe ingredients. Manufacturers are required to ensure the safety of new ingredients [44]. They are required to notify the FDA ≥75 d before introducing NDI into interstate commerce and must submit supporting evidence that the NDI is expected to be safe.

Labeling requirements for supplements are similar to foods, with a few differences [45]. The statement of identity must be “dietary supplement” with the flexibility to omit or change the term “dietary” to alternate words such as herbal. Instead of a nutrition facts panel, dietary supplements have a “Supplement Facts” panel. Labels include net content, an ingredient list, direction of use, name and place of the business, claims, and any cautionary and/or warning statements.

#### Supplement benefits and claims

Most dietary supplements include claims regarding the relationship between the dietary ingredient and a benefit. Claims may include structure-function, general well-being, and nutrient deficiency claims, which are referred to as 403(r)(6) claims [46]. The FDA requires manufacturers to notify them of these claims no later than 30 d after marketing the supplement. The FDA also requires the claim to be accompanied by the statement, “This statement has not been evaluated by the FDA. This product is not intended to diagnose, treat, cure, or prevent any disease,” on the label or labeling wherever the claim is made [47]. If a supplement manufacturer makes a drug claim that diagnoses, mitigates, treats, cures, or prevents disease, the FDA issues courtesy letters or warning letters notifying the manufacturer that their product does not meet the definition of a dietary supplement. Examples of where the FDA has responded to drug-related claims include statements that the supplement promotes healthier blood sugar concentrations, which implies diabetes or may reduce cholesterol, which implies heart disease.

#### Personalized supplements

In 2023, the *Nutrition Business Journal* reported [48] that sales of personalized supplements would reach $1.15 billion at the end of 2024, which was 15 times the estimate in 2017. Personalized supplement companies often use surveys or questionnaires to identify and characterize user recommendations. Other measures such as genetics or blood testing or information from wearable devices, like fitness trackers, may also help inform recommendations. Messaging to the individual must not use language that suggests the supplement prevents, cures or mitigates a disease (e.g., Do you have a history of urinary tract infections?). Health- and wellness-focused questions, such as “Are you interested in urinary tract health?” can be used to help provide tailored recommendations within the current regulatory framework.

#### Personalized food and supplement small package labeling

The present regulatory framework defines packaging and labeling requirements, including specific provisions for small packages [45,49,50]. Appropriate packaging can be an opportunity to provide information to the user and ensure transparency while maintaining label and font requirements. For example, personalized supplements in individual sachets or packets have limited space for labeling and are often accompanied by an insert, handout, or pamphlet to meet the labeling requirements. Safety-related information, such as warning and allergen statements, may be printed on each packet to ensure the consumer is aware of the safety information in the absence of the accompanying insert.

#### PN, dietary plans and patterns, and government food policy

The DGAs, which are updated every 5 y, provide population advice on what to eat and drink to meet nutrient needs, promote health, and help prevent chronic diseases [51]. The guidelines are central to the health of the general population and are not individualized. Likewise, dietary reference intakes (DRIs), established by the National Academy of Sciences, Engineering, and Medicine, are population estimates of nutrient intakes to be used for planning and assessing diets applicable to healthy people [52]. The DRIs have guidance based on the age and sex of the population but do not specifically focus on the individual.

Numerous types of diets recommended by health professionals offer a degree of tailoring for specific needs and preferences that fit within the DGA. These dietary patterns can include a Mediterranean diet, Dietary Approaches to Stop Hypertension diet, vegetarian or vegan diet, low carbohydrate/high fiber diet, etc. Each of these diets can be modified to help address cultural or other preferences such as sustainability. Personalized dietary patterns are not currently incorporated in any government regulations apart from the FDA assuring approval has been secured for the use of an in vitro diagnostics (IVD), medical device, or an authorized or qualified health claim. If a claim is made that a diet is beneficial for a specific outcome, whether it is weight loss, reducing blood sugar, or hemoglobin A1c, scientific evidence is required to support the claim. It has been suggested that diets for individuals with diabetes should be considered within the medical food category because some may be formulated to be low in carbohydrates, high in dietary fiber, and often moderate in fat content. The FDA states [24] that diabetes is not a category for which medical foods are indicated because a regular diet can be modified to meet the dietary requirements for a diabetic diet [53].

In contrast to the population-oriented DGAs and DRIs, which provide recommendations by age and sex, PN offers an innovative approach to what an individual might need or want to achieve health and functional goals beyond chronic disease prevention. PN can be tailored to promote the health and wellness of the individual and may integrate demographic, genotypic, phenotypic, social, environmental, cultural, and behavioral factors to inform recommendations. The integration of these factors enables health professionals, including RDNs, to recommend types and quantities of food and dietary supplements, as well as activities, where appropriate.

#### Medical device development

Currently, many PN programs include medical devices, and this number will likely expand. Understanding the regulatory framework for medical device development is necessary for PN programs that will incorporate and/or develop new technologies, such as wearable devices (e.g., nutrient-sensing sweat patches), software applications, or AI, to enable current applications. Medical devices may be incorporated into the PN system as tools to support data collection and analysis that provide context into an individual’s lifestyle behaviors (Figure 2). Medical devices [54] include software as a medical device (SaMD), IVD, instruments, apparatuses, implements, machines, other contrivances, or implants that are intended to either diagnose, cure, mitigate, treat, or even prevent diseases from occurring in humans or animals. Medical devices are regulated by the FDA’s Center for Devices and Radiological Health (CDRH). Products that achieve their intended purpose through chemical action (e.g., analgesics, antibiotics) are drugs or biologics and, thus, not regulated by the CDRH.

**FIGURE 4:**
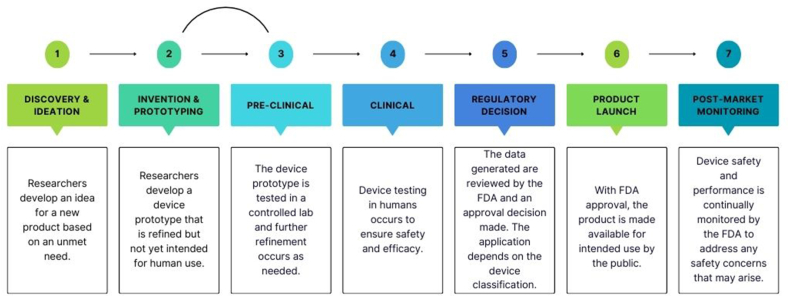
The medical device development pathway. This schematic is an expanded representation of the device development process as established by the FDA. FDA, Food and Drug Administration. Created using canva.com.

Establishing a clear application and intended use for a new medical device is critical to aligning with regulatory requirements and ensuring the collection of comprehensive evidence since the development process (Figure 4) requires a substantial investment of time (∼3–7 y) and money ($5–$150 million or more) [55,56]. Clear regulatory, manufacturing, and clinical strategies, including a design development plan, need to be in place to ensure all the required information is gathered for approval of the device. It is important to have a detailed product profile. This serves as a communication tool between the FDA, team members, investors, and consultants, and a framework that can be updated in real-time and used as the labeling for the device in development.

#### Regulation of medical devices

Devices used for PN programs may not all be considered medical devices (e.g., actigraph or movement tracker). The FDA’s “General Wellness: Policy for Low-Risk Devices” guidance clarifies the regulatory approach for general wellness devices that promote healthy lifestyles and present a low risk to users [57]. The document outlines criteria to define these devices, emphasizing that they should either encourage general health or support a healthy lifestyle’s role in managing chronic conditions without diagnostic or therapeutic claims. Low-risk general wellness devices are typically exempt from regulatory requirements, provided they are noninvasive, nonimplanted, and use safe technology. Examples include fitness trackers, relaxation applications, and noninvasive cosmetic tools.

Some PN programs may incorporate regulated medical devices [e.g., continuous glucose monitor (CGM)]. Thus, an overview of FDA medical device regulation is included here. The FDA regulates and approves medical devices by classification I-III, categorized from least to greatest risk to the user (Table 2) [54,58,59], intended use, and indication for use [60]. Class I devices are the least risky devices and merely require registration with the FDA. Class II devices are medium risk and may show equivalence to previously approved class II devices through a premarket 510(k) clearance submission to the FDA. The FDA has 90 d to evaluate the 510(k)-clearance submission and can extend this period if questions arise, making it critical to promptly respond to questions to minimize delays. At the end of the 90 d, the FDA can either reject the application or allow approval. De novo applications are required if no previously approved class II devices exist. A de novo application requires demonstrated safety and efficacy of the device to ensure it meets the quality level required of a class II device. It may include validation, preclinical testing, and clinical studies to demonstrate safety and efficacy. Information on identifying class II devices and if predicate devices exist can be found within the medical device databases on the FDA’s website [61]. Class III is the greatest risk, and, in the case of IVD, it involves contact with the user, which can influence the data output. Class III devices require the greatest amount of information for approval, which occurs through a premarket approval process. The premarket approval requires additional validation data in the application, such as nonclinical laboratory and animal testing. Also, more extensive clinical studies are required to approve the device. The FDA provides definitions of this through guidance documents on the FDA’s website [62].

**TABLE 2:** Classifications of medical devices and in vitro diagnostics.

Classification	Description	User risk	Medical device^1^ example	IVD^2^ example	SaMD^3^ example
Class I	These low-risk devices are often exempt from review and may merely require registration with the FDA.	Low	Bandage	Photometric method for ammonia detection in a device	Retrospective clinical data analysis device
Class II	These devices may require showing equivalence to previously approved class II devices under a 510(k) review but do not usually require clinical trials.	Medium	Infusion pump	Method for hemoglobin A1c detection	Dexcom G7 CGM
Class III	These devices need the greatest amount of information for approval, which occurs through the Premarket Approval process. These devices require extensive safety and efficacy clinical trial data to show that risk to the user can be minimized.	High	Pacemaker	Invasive glucose sensor	Guardian connect CGM

Abbreviations: CGM, continuous glucose monitor; FDA, Food and Drug Administration; FDAC, Food, Drug, and Cosmetic Act; IMDRF, International Medical Device Regulators Forum; IVD, in vitro diagnostics; SaMD, software as a medical device.

1 As defined by the FDAC, medical devices [54] are instruments, apparatuses, implements, machines, other contrivances, or implants that are intended to diagnose, cure, mitigate, treat, or even prevent diseases from occurring in humans or animals.

2 As defined by the FDAC, IVD [58] are those reagents, instruments, and systems intended for use in the diagnosis of conditions or health states in order to cure, mitigate, treat, or prevent disease.

3 As defined by IMDRF [59], “software intended to be used for 1 or more medical purposes that perform these purposes without being part of a hardware medical device.”

#### IVD quality monitoring

PN companies may seek to utilize regulated IVD tests (e.g., ovulation tests) as part of their program offering. An important consideration in the IVD regulatory strategy is how the device will be used and how the quality of the results will be maintained. Adhering to clinical laboratory improvement amendments (CLIA) [63] is important for the regulatory strategy, and agreement is required from the FDA before device development begins. CLIA [64] are federal standards applicable to sites that test human samples for a health or disease assessment (e.g., pulse oximetry). Tests where there is little risk of error are waived from CLIA law. Examples of CLIA-waived tests [65] include blood glucose, cholesterol, and hemoglobin A1c. The FDA is responsible for determining the IVD complexity category (waived, moderate, or high complexity test product). A waived test means no additional testing is done. For example, home diagnostic tests are generally waived tests and do not require quality monitoring throughout use. Moderate complexity tests require clinical laboratories to analyze tests on a regular basis, meet all the quality standards, and provide appropriate results across different clinical laboratories. High-complexity tests require even more frequent demonstration of quality (e.g., molecular diagnostic testing). Tests of all different complexity classifications are used in PN programs. Therefore, understanding the application of the complexity categories prior to the development of these programs is critical to support user safety and evidence-based outcomes.

#### Medical device development support

Support for medical device strategy and development can be sought in several ways (Table 3). The CDRH website [66] has a wealth of information in databases, including guidance documents, videos, news, and information that can be used during the development of a medical device. Subject matter experts, advisory boards, and entrepreneurs in residence can help improve various medical device and IVD development strategies. The International Standards Organization and the American Society for Testing and Materials International have a series of guidance documents on different aspects of medical device development. Further, the FDA website has useful guidance documents, videos, and presentations by FDA reviewers and outside experts on various pertinent topics. The FDA encourages communication early and often about presubmission information in the form of a proposal through the Q-submission program. This program allows presubmission feedback, issue requests, study risk determinations, or general information requests on strategy to ensure clarity on regulatory expectations and a smooth review process [67]. The FDA responds to focused Q proposals with nonbinding feedback.

**TABLE 3:** International medical device regulators forum software as medical devise risk levels and how they are evaluated.

SaMD criticality^2^	Treat or diagnose^1^	Drive clinical management^1^	Inform clinical management^1^
	Risk level I-IV^3^		
Critical	IV	III	II
Serious	III	II	I
Nonserious	II	I	I

Abbreviations: IMDRF, international medical device regulators forum; SaMD, software as a medical device.

1 Significance of information provided by the SaMD to the healthcare decision.

2 The IMDRF evaluated different applications for SaMD and evaluated the criticality of the device to the state of the healthcare situation/condition for the user.

3 Risk levels are categorized from the least risk (I) to the greatest risk (IV) to the user.

#### Wearables and devices

The development and use of wearable devices is rapidly growing. A recent study of 3000 United States individuals interested in PN showed that 45% use a fitness tracker at least once a week [68]. Wearable devices need to be reliable and accurate, and regulatory considerations are needed regarding how users’ data are deployed. If medical data are collected and intended for use in the diagnosis of disease or other conditions or in the cure, mitigation, treatment, or prevention of disease, then the wearable may be considered a medical device [69]. If the medically related data are excluded from the collection (e.g., atrial fibrillation data), exercise and fitness tracker data can enrich the ability to tailor lifestyle recommendations. The FDA provides guidance [54] to help determine if a wearable or other device should be considered a medical device. However, there are some areas of ambiguity that need to be considered. For example, a device (e.g., Apple Watch) can be used to track the menstrual cycle to identify insights and correlations between the menstrual cycle and fertility. If a physician is using a wearable device for healthcare, the Apple Watch can be seen as a medical device. If the data from the Apple Watch are also being used to inform on infertility or polycystic ovary syndrome, the nutritional recommendations provided are considered MNT and require a physician referral and implementation by RDNs. There is also an opportunity to support practitioners in training on the integration and use of wearables to support equitable health outcomes [[70], [71], [72], [73], [74]].

#### Application of the regulatory framework for PN

**FIGURE 5:**
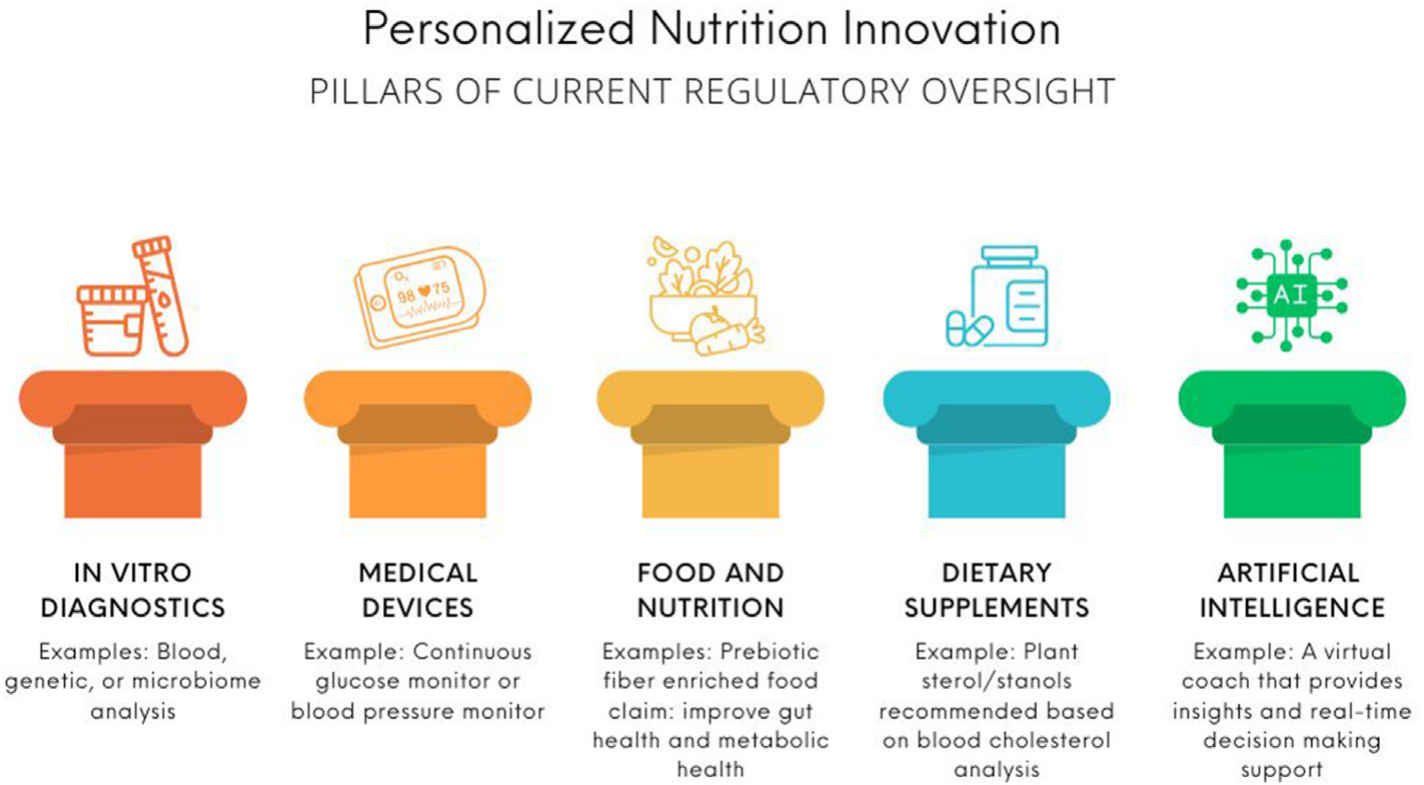
The pillars of current regulatory oversight in personalized nutrition. This schematic visualizes well-defined areas of the current United States regulatory framework and examples of how different components would be regulated. As the field is rapidly developing, the application of new technology may require updates to the regulatory framework. Created using canva.com.

Companies that provide PN programs need to ensure they have met all the different regulatory criteria based on what they are providing to the user (Figure 5). The following will highlight a hypothetical PN company to exemplify how different regulations apply in the PN program. The company’s program includes an at-home test kit for blood lipids (Figure 5, IVD pillar) and a CGM device (Figure 5, medical device pillar) to deliver PN recommendations for overweight and obese individuals and people with prediabetes. The CGM is worn for 2 wk, and it aggregates glucose concentrations in terms of the individual’s responses to food and physical activity. The user can scan barcodes on foods for predictive responses to consumption. The system can provide alternative choices and behavior change guidance via an AI-driven virtual coach developed by qualified health professionals to optimize glucose concentrations (Figure 5, AI pillar). In addition, both a functional food with a substantiated claim to improve gut and metabolic health (Figure 5, food and nutrition pillar) and a plant sterol/stanol supplement (Figure 5**,** dietary supplements pillar) with an authorized health claim to help reduce cholesterol is recommended. It is appropriate to recommend lifestyle changes to the overweight population and those with prediabetes. People with diabetes and those with morbid obesity have metabolic disorders requiring RDN and physician oversight and guidance in developing any algorithms, training the AI, and developing appropriate recommendations. Also, scientifically substantiated evidence is required to meet FDA and FTC regulations for any claim that a food conveys a health benefit [22].

#### AI overview

Significant advancements and the adoption of AI in the PN system have occurred. AI and ML via virtual chatbots can provide individuals with real-time lifestyle insights. Using AI responsibly necessitates understanding questions such as, “What are the credentials of recommendation algorithm developers?,” “Who is overseeing the substantiation of algorithms?” and “What datasets are being used to train the AI?” AI and ML models used in PN must be developed and validated under the supervision of credentialed health professionals to ensure accuracy and validity. The datasets used to train these models should be diverse and representative to avoid biases. AI-driven PN solutions require transparency in how algorithms are developed, validated, and used. Clear communication about the benefits and limitations of AI-driven recommendations is essential to gain user trust and engagement. Integrating AI and ML in PN programs raises questions about data privacy, security, and ethical use. Regulatory bodies need to establish guidelines for the responsible use of these technologies, including avoiding algorithmic discrimination and protecting user data.

A blueprint for an AI bill of rights has been developed by the Office of Science and Technology Policy [75]. It does not provide specific criteria relative to nutrition. However, it does cover a regulatory framework that provides ethical guidelines focusing on responsible design and use of AI, the avoidance of algorithmic discrimination, and data protection. A recent perspective specifically addresses PN (Abrahams et al. under review). It notes the potential of AI to increase PN access and affordability. It also calls for the need to maintain human oversight and collaboration of multiple stakeholders in nutrition, behavior, and regulation, as well as responsible training and use of AI. This will ensure logical, available, and ethical solutions to build trust and credibility in the field. In addition, advancing and collecting longitudinal data showing the efficacy of PN [76] will also help solidify the value of the model. The ethics of AI, the AI Bill of Rights, and the international focus on AI regulations highlight the increasing importance of regulations in this area.

#### AI or ML SaMD

AI and ML are technologies that are now being incorporated into medical devices, and they are called SaMD. SaMD is intended to treat, diagnose, cure, mitigate, and prevent diseases. Examples of SaMD include patient portals, telemedicine healthcare provider portals, and potentially health trackers (if used for this purpose). SaMD functions independently and does not include software that is integral to the operation of hardware medical devices, which would be regulated with that hardware. The CDRH is the agency within the FDA that regulates SaMD. However, since this is a relatively new area, the CDRH partnered with global regulatory agencies to create the international medical device regulators forum (IMDRF). IMDRF created risk levels I-IV for SaMD, categorized by the least to the greatest risk to the user. These categorizations are used to view different applications for SaMD, evaluate the criticality of the device, and label it as critical, serious, or nonserious. The IMDRF also assesses the different applications for these devices to create a grid of risk levels and how those risk levels are evaluated (Table 3). They also created a quality management system with a common understanding of vocabulary for the various applications. The quality system describes how the risk categorization affects the quality control and evaluation from a clinical and technical perspective of the device. It demonstrates the safety, efficacy, and performance regarding the valid clinical association, analytical and clinical validation. Additional information can be found on the FDA’s website [77], including SaMD’s database. Like all medical devices, SaMD is approved based on classification I-III, categorized by the least to the greatest risk to the user (Table 2) [54,58,59], intended use, and indication for use.

#### Privacy and integration of third parties

Data privacy is a critical feature in PN programs and builds user trust. In 2018, the European Union implemented regulations, such as general data protection regulations, to protect the information of residents. General data protection regulation applies to all companies that process the personal data of Europeans regardless of where the company is based [78]. Currently, 17 states in the United States have similar privacy laws [79], and regulators are paying attention to personal data linked to PN recommendations. Recently, a company was fined for failing to protect its customers’ genetic data [80]. Another data breach was cited with 23andMe in 2023 and resulted in changes to 23andMe actions and privacy policies [81]. These demonstrate that regulators are committed to ensuring users’ data are protected and that truthful information on the use of their personal data are received.

Because PN programs often need an ecosystem and a group of collaborators to develop multi-component programs to support the customers’ needs, there is an increasing number of partnerships between companies. For example, companies are partnering to deliver dietary intake tracking capabilities, personalized recipe platforms, and recommendations on shopping and exercise. Partnerships add another layer of privacy considerations to ensure data are as secure as possible when shared between parties and feedback delivered to the user. Data sharing on platforms needs to be transparent, and data use agreements should be understood by an audience that has no special or expert knowledge. How data are used and shared must be based on what the user understood and agreed upon when they signed up. Communicating results and recommendations to the user must ensure privacy. Texts, telehealth platforms, and emails all need to be highly secure. Coaching services need to be on a secure and encrypted platform. Platforms must protect users’ data by preventing information leaks.

### Navigating oversight complexities and the future horizon in PN

#### The complexities of regulating PN programs

Individuals have unique circumstances (genetics, epigenetics, and exposome factors), which result in the need for multiple components in PN programs to be able to tailor them to the individual. Therefore, there will be many components rather than a single intervention making up the PN program. This may include tools to support a variety of biological measures, behavioral, social, environmental, and other elements. Therefore, a comprehensive regulatory framework to oversee all elements of PN may not be feasible or necessary. The primary focus of the FDA is to ensure safety and promote health. Likewise, the focus of regulating PN should be on ensuring the tools, advice, and choices provided to users are safe, free of difficulties, and completed under trained professionals’ supervision (e.g., dietitians and other healthcare professionals). In this regard, the various components of the PN program, including various interventions, can operate within existing regulations. However, this does not preclude the potential need for an evolving regulatory framework for components with new innovations.

With the rapid development of software, wearables, and other devices, it can be difficult to distinguish between medical devices and applications for general wellness. The PN industry should be at the forefront of creating and implementing guidance on safe and effective programs and approaches in partnership with the FDA. Encouraging companies to communicate early with the FDA could also make a difference in user acceptability, trust, and engagement.

#### Navigating regulations while supporting PN claims

As discussed above, disease-state claims on and about PN programs cannot be made. PN programs intended for the general population cannot state they treat, diagnose, mitigate, prevent, or cure diseases. Sometimes, PN programs will incorporate testing of biomarkers that may also be used when collecting medical information. For example, suppose a PN program tests for LDL cholesterol. The users can be informed that LDL cholesterol is 1 marker of cardiovascular health. For food, the PN program could inform users it has been shown to help maintain LDL cholesterol, which supports heart health (provided the product meets all requirements for this statement). A health claim would be required if users were informed that the food reduces the risk of cardiovascular disease. It should be noted that the FDA and/or the FTC will step in when unauthorized claims are false or misleading, or the health, safety, or privacy of individuals are at risk, which was recently exemplified by several genetic testing companies that were providing diagnostic advice [82].

#### Challenges, opportunities, and potential solutions for the future of PN

PN programs should deliver a health or functional benefit; however, these programs are often marketed without prior research to evaluate their effectiveness. Developing an evidence base for a PN program should not rely solely on secondary data sources. Substantiation of benefits through clinical research demonstrating efficacy is essential. This can be an opportunity for program differentiation and to support new, responsible, transparent, and evidenced-based claims and communications. Controlled trials are needed to understand when and where better outcomes are achieved through PN interventions relative to general population-based guidelines. Because the established biomarkers are related to diseases such as diabetes or cardiovascular disease, many studies demonstrating the benefits of personalized approaches have focused on these areas [5,9,83]. However, in other areas, noninvasive biomarkers of health, well-being, and physical and mental performance need to be identified and evaluated to provide the opportunity to limit or avoid safety risks to the end user and align with their needs.

The regulatory environment for PN programs must evolve with this rapidly advancing market to ensure the delivery of safe, effective, substantiated, clearly communicated, and accessible solutions. PN accessibility can be difficult for underrepresented groups and compounded by challenges such as lifestyle choices, chronic disease, limited food access, food insecurity, and disordered eating [84,85]. These challenges highlight important opportunities to support the democratization of PN to provide substantiated benefits and help improve choices. To accomplish this goal, there is a need to ensure that data sets are representative so that solutions can be effective. This information includes biological and behavioral data sets.

Another critical factor in the efficacy of PN programs is incorporating behavioral science to support meaningful communication with PN users. Recommendations need to fit easily into the users’ day-to-day activities and be provided at the right time. This may require the delivery of information via a technological device. These requirements become important when considering technology integration and the use of AI systems because there is the potential for misinformation [86]. There is the potential to do harm, so it is essential to have the right experts to structure the data models appropriately.

Overall, awareness and understanding within the PN industry of the regulations that apply in this rapidly changing area is limited. There is an opportunity to set up a network of individuals with expertise and experience with PN and regulations to provide guidance as more companies enter this space, maybe even some type of certification or seal to communicate a level of quality to the user. This would provide innovative startups as well as larger companies interested in participating in the PN programs access to learn which regulations apply, what they should have in place, and what they are allowed to claim. In addition, this network would support companies’ understanding of implications for data security relative to partnerships, developing application programming interfaces, and data sharing. It is critical to look at the present environment, its history, and the long-term regulatory implications, including limitations.

In summary, PN programs require several components that may need to be regulated differently and unique and collaborative support, including credentialed and skilled health professionals (e.g., RDNs, physicians, nurses, psychologists, behavioralists, health and wellness coaches, and other clinicians, etc.) depending on the application. For companies practicing or selling PN, working closely with regulatory bodies to support privacy, transparency, and substantiation of benefits is an opportunity and source of differentiation. The current system allows the PN providers to communicate with users regarding wellness and maintenance of health, but there is currently no regulatory path to communicate about disease prevention. This is critical given deaths from chronic disease are on the rise and account for 60% of deaths worldwide, where lifestyle modification can be the most controllable and influential factor [[87], [88], [89]]. Although PN can operate within the current regulatory framework, the field is new and rapidly changing, which necessitates modifications to current regulations. Global collaboration with regulatory bodies, such as the European Union Data Protection Board and the IMDRF, can help bolster and harmonize standards and facilitate the global adoption of innovations while ensuring the safety, oversight, and efficacy of PN programs, including factual and transparent communication of benefits. As these regulations are developing, the opportunity exists to form independent organizations or networks to provide guidance. This could include support for understanding regulatory requirements for innovation and working with policymakers and government bodies. The possibility exists to provide specific training and certification in the regulation of PN programs.

## Author contributions

The authors’ responsibilities were as follows – SMD, A-SK: directed the workshop; A-SK, BLW, JCA, KMN, SMD: developed the concept for the manuscript; BLW, JCA, KMN: led manuscript development; MA, GB, EGM, TAM, TW: were speakers during the workshop from which the manuscript content was based upon. The views expressed are those of the authors and do not necessarily reflect the position or policy of their affiliate companies/organizations. All authors contributed to the manuscript development; and all authors: read and approved the final manuscript.

## Funding

This session was funded by the Personalized Nutrition Initiative at the University of Illinois Urbana-Champaign through internal funds and External partner program membership fees. The 2023–2024 External Partner program members were Amway, Archer Daniels Midland Co, General Mills, Givaudan, Mars Wrigley, National Dairy Council, PepsiCo, and Pharmavite. Representatives from the External Partners contributed to planning the agenda and identifying speakers, participating in the live session, and reviewing the manuscript.

## Conflict of interest

KMN reports financial support was provided by University of Illinois Urbana-Champaign. BLW reports financial support was provided by University of Illinois Urbana-Champaign. JCA reports financial support was provided by University of Illinois Urbana-Champaign; reports a relationship with University of Illinois Urbana-Champaign that includes: consulting or advisory, speaking and lecture fees, and travel reimbursement; reports a relationship with Dairy Management Inc. that includes: consulting or advisory and travel reimbursement; reports a relationship with Shaklee that includes: consulting or advisory and travel reimbursement; reports a relationship with GOED that includes: consulting or advisory; reports a relationship with Brightseed that includes: consulting or advisory and travel reimbursement; reports a relationship with Ocean Spray Cranberries Inc that includes: consulting or advisory and travel reimbursement; reports a relationship with Bobbie that includes: consulting or advisory and travel reimbursement; reports a relationship with Juvenescence that includes: consulting or advisory; reports a relationship with McCormick that includes: consulting or advisory. GB reports a relationship with University of Illinois Urbana-Champaign that includes: speaking and lecture fees and travel reimbursement; reports a relationship with Segterra Inc that includes: board membership, employment, and equity or stocks. SMD reports a relationship with General Mills Inc that includes: consulting or advisory and speaking and lecture fees; reports a relationship with PepsiCo Inc that includes: consulting or advisory and speaking and lecture fees. TAM reports a relationship with University of Illinois Urbana-Champaign that includes: speaking and lecture fees. MA reports a relationship with University of Illinois Urbana-Champaign that includes: consulting or advisory, speaking and lecture fees, and travel reimbursement. GB has patent #US8762167B2 pending to Segterra Inc. JA, TB, GB, MA, and RB External Advisory Committee member for the Personalized. Nutrition Initiative at the University of Illinois Urbana-Champaign. SMD is Director of the Personalized Nutrition Initiative at the University of Illinois at Urbana-Champaign. A-SK is Assistant Director of the Personalized Nutrition Initiative at the University of Illinois at Urbana-Champaign.

JCA, BLW, and KMN: works at Nlumn; the company that got compensated by the University of Illinois at Urbana-Champaign to draft the manuscript based on the workshop presentations and discussions. The funds from this compensation came from the External Partner Program membership fees. The workshop was funded by the Personalized Nutrition Initiative at the University of Illinois Urbana-Champaign through internal funds and External Partner Program membership fees. Representatives from the External Partners contributed to planning the agenda and identifying speakers, participating in the live session, and reviewing the manuscript.

RB is currently employed by ADM. TDB is currently employed by PepsiCo. TW is currently employed by Pharmavite. AS is currently an employee of the Academy of Nutrition and Dietetics which supports RDNs. JMO has previously been the associate editor of Advances in Nutrition. If there are other authors, they declare that they have no known competing financial interests or personal relationships that could have appeared to influence the work reported in this article.
